# The structural insights of stem cell factor receptor (c-Kit) interaction with tyrosine phosphatase-2 (Shp-2): An *in silico *analysis

**DOI:** 10.1186/1756-0500-3-14

**Published:** 2010-01-22

**Authors:** Soumya Pati, Gangenahalli U Gurudutta, Om P Kalra, Asok Mukhopadhyay

**Affiliations:** 1 Stem Cell Biology Laboratory, National Institute of Immunology, New Delhi, India; 2 Stem Cell and Gene Therapy Research Lab, Institute of Nuclear Medicine and Allied Sciences, Delhi, India; 3 Department of Medicine, University College of Medical Sciences & Guru Teg Bahadur Hospital, University of Delhi, New Delhi, India; 4 Gene Regulation Laboratory, National Institute of Immunology, New Delhi, India; 5 Current Address: Department of Neuroscience, School of Medical Sciences, University Sains Malaysia, Malaysia

## Abstract

**Background:**

Stem cell factor (SCF) receptor c-Kit is recognized as a key signaling molecule, which transduces signals for the proliferation, differentiation and survival of stem cells. Binding of SCF to its receptor triggers transactivation, leading to the recruitment of kinases and phosphatases to the docking platforms of c-Kit catalytic domain. Tyrosine phosphatase-1 (Shp-1) deactivates/attenuates 'Kit' kinase activity. Whereas, Asp816Val mutation in the Kit activation loop transforms kinase domain to a constitutively activated state (switch off-to-on state), in a ligand-independent manner. This phenomenon completely abrogates negative regulation of Shp-1. To predict the possible molecular basis of interaction between c-Kit and Shp-1, we have performed an *in silico *protein-protein docking study between crystal structure of activated c-Kit (phosphorylated c-Kit) and full length crystal structure of Shp-2, a close structural counterpart of Shp-1.

**Findings:**

Study revealed a stretch of conserved amino acids (Lys818 to Ser821) in the Kit activation domain, which makes decisive H-bonds with N-sh2 and phosphotyrosine binding pocket residues of the phosphatase. These H-bonds may impose an inhibitory steric hindrance to the catalytic domain of c-Kit, there by blocking further interaction of the activation loop molecules with incoming kinases. We have also predicted a phosphotyrosine binding pocket in SH2 domains of Shp-1, which is found to be predominantly closer to a catalytic groove like structure in c-Kit kinase domain.

**Conclusions:**

This study predicts that crucial hydrogen bonding between N-sh2 domain of Shp-1 and Kit activation loop can modulate the negative regulation of c-Kit kinase by Shp-1. Thus, this finding is expected to play a significant role in designing suitable gain-of-function c-Kit mutants for inducing conditional proliferation of hematopoietic stem cells.

## Findings

### Research hypothesis

The c-Kit controls major signaling cascades in hematopoietic stem cells. Earlier study indicated that the catalytic domain of c-Kit consists of many important loop regions, such as catalytic loop (786-796), activation loop (810-839) and substrate-binding loop (829-837), which play crucial role in the activation of Kit kinase domain [1]. Transformation of c-Kit kinase domain from an inactive to hyperactive state is reported to occur on the basis of three distinct molecular phenomena. These are (i) binding of ATP to glycine rich loop residues (596-601), (ii) release of self-inhibitory interaction between substrate binding loop and activation loop, and (iii) loss of intramolecular H-bonds between Lys818-Asp816 and Asn819-Asp816 [1].

Binding of stem cell factor (SCF) to its receptors triggers dimerization coupled transphosphorylation of c-Kit catalytic domain [2-4], which then emerges with a dual phosphorylated tyrosine containing conserved motif (Y^568^V^569^Y^570^) in human c-Kit [5]. Interestingly, this motif acts as a common docking site for SH2 and SH3 domain containing proteins [6]. The adaptor protein APS, Src family of kinases and Shp-2 tyrosine phosphatase bind to Y^568^. Similarly, Shp-1 tyrosine phosphatase and the adaptor protein Shc bind to Y^570^; whereas, C-terminal Src homologous kinase (Chk) and the adaptor Shc bind to both Y^568 ^and Y^570 ^[7]. Shp-1 is known as a negative regulator of various receptors in hematopoietic cells, including c-Kit kinase [8,9]. Shp-1 and Shp-2 share both sequence and structural homologies [10]. Previous studies have shown that a gain of function mutation, Asp816 to Val816 (a molecular representative of mastocytosis) in Kit activation loop leads to extensive degradation of Shp-1, indicating disruption of Shp-1 and c-Kit interaction [11,12].

Though SH2 domains of both Shp-1 and Shp-2 are known to interact with c-Kit, the molecular basis of this interaction has not been elucidated. The present study suggests some important residues in Kit activation loop and N-sh2 domain that play crucial role in negative regulation of c-Kit kinase.

## Results

### Multiple sequence alignment of c-Kit kinase related protein

**Figure 1:**
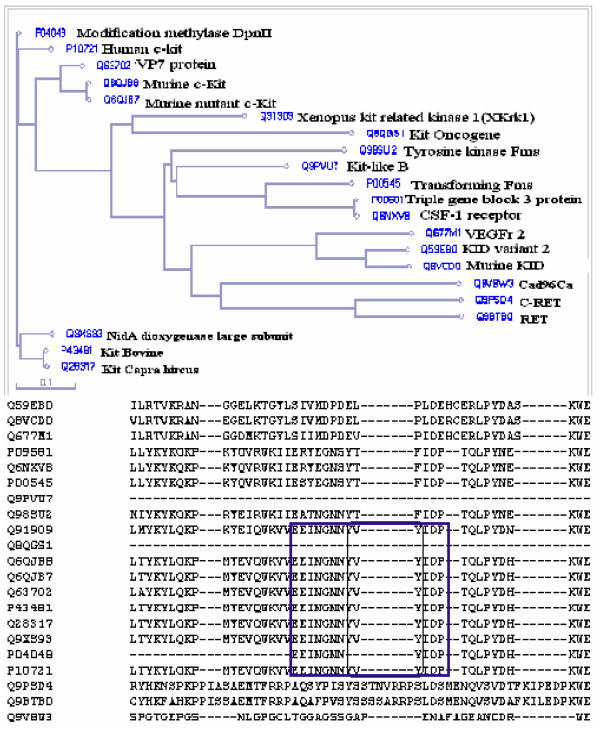
**Multiple sequence alignment of c-kit juxtamembrane domain. Multiple sequence alignment in combination with phylogenetic tree was generated by PIR-ALN for Kit kinase with other kinase family members**. Alignments show dual tyrosine containing conserved Y^568^V^569^Y^570 ^stretch of residues in "EEINGNNYVYIDP" motif, which is used as a docking platform for SH2 and SH3 domain containing proteins.

Activated Kit kinase, triggered either by ligand-dependent or ligand-independent manner, generates several phosphorylated tyrosine containing docking motifs in its juxtamembrane domain. Multiple sequence alignment study showed a conserved regulatory motif "EEINGNNYVYIDP" in juxtamembrane domain of Kit kinase (Fig. 1). Further analysis of "EEINGNNYVYIDP" by Motif scan showed a conserved dual tyrosine residue, which is positioned as Y^568^V^569^Y^570 ^in human c-Kit (Additional file 1). These two tyrosine residues, Y^568 ^and Y^570 ^at positions +1 and +3 act as docking molecule for various kinases as well as SH2 and SH3 domain containing proteins. Amongst SH2 and SH3 domain containing proteins epidermal growth factor receptor, platelet derived growth factor receptor, PLCg, N/C-sh2, Abl kinase, Src family kinases and phosphatases (Shp-1 and Shp-2) are important [13-15]. The alignment study also revealed deletion in the above motif (YV deleted at +1, +2 positions) in transforming tyrosine-protein kinase Kit (Swiss prot ID P04048), normally synthesized as Gag-Kit-Pol polyprotein. The crystal structure data of c-Kit also expose Y^568 ^and Y^570 ^as the primary dual tyrosines to get phosphorylated following *in vitro *trans-phosphorylation reaction [5]. So they predominantly act as primary docking site *in vivo *to the downstream signaling molecules. These two residues belong to juxtamembrane switch motif of c-Kit (stretched in 560-571 residues) and is also a part of auto inhibitory JMD (spanning from 553 to 663), which makes a putative α helix. This juxtamembrane segment in c-Kit makes a wedge that stabilizes inactive conformation by preventing rotation of the small kinase lobe towards the large kinase lobe to generate active kinase conformation. Point mutations in this motif are associated with gastrointestinal stromal tumors, as they abolish the regulation of Kit kinase activity [16,17]. In Gag-Kit the deletion of "YV" in "EEINGNNYVYIDP" motif and other deleted auto-inhibitory JMD residues before the same motif suggest two possible reasons for its transformation. The first reason could be due to the abrogation of Shp-1 mediated negative regulation, as Shp-1 can not be recruited to YVY. The second reason may be due to change of JMD mediated auto-inhibition of kit kinase to its hyper-activated state [18-20].

### Multiple sequence alignment of the ligand: Shp-2 and Shp-1 with SH2 domain containing proteins

**Figure 2:**
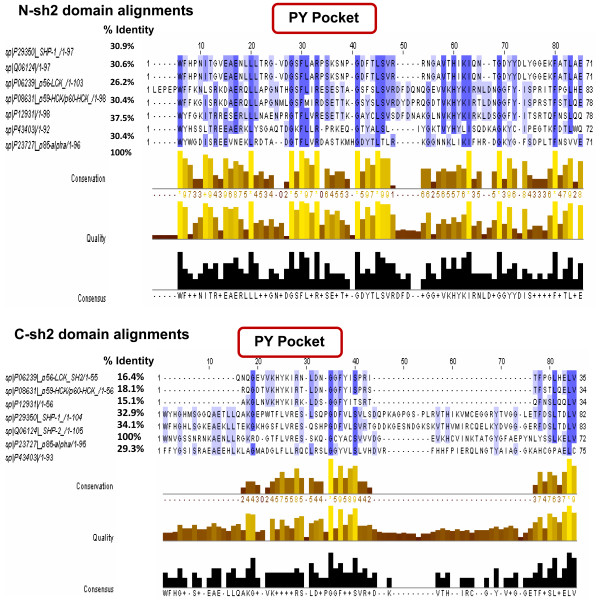
**Multiple sequence alignment of SH2 domains of Shp-1 and Shp-2 for analysis of PY pocket residues**. SH2 domain alignment show conserved PY pockets, such as "FLVRES" in C-sh2 and "FLARPS" in N-sh2 domains of both Shp-1 and Shp-2. Percentage of sequence identity, conservation of residues in PY pockets as well as sh2 domains are shown as change in color intensity. PY pocket is found to be highly conserved in N-sh2 domains, in comparison to C-sh2 domains.

To identify the crucial residues in Shp-2 and Shp-1 that can interact with tyrosine kinases, multiple alignments of SH2 domains of phosphatases with other SH2 domain family proteins was performed. The SH2 domains of p85 subunit of PI3 kinase, LCK, HCK, Src and c-ZAP showed conserved phosphorylated tyrosine-binding pockets (PY pockets) comprising of "FLARPS" in N-sh2 and "FLVRES" in C-sh2 domains phosphatases (Fig. 2). PY pockets represented three fully conserved amino acid residues: F, L and R. The functionally important residues in this motif initiate strong decisive interactions between guanidinium group of invariant arginine residue at a conserved position in SH2 and anionic PY phosphate group, as shown in structure of p85 subunit of PI3 kinase [21]. This PY pocket showed 100% conservation of Phe and Arg at +1 and +4 positions, 85% conservation of Leu at +2 and 71% conservation of Ser at +6 positions, respectively in N-sh2 domain containing proteins (Fig. 2). This explains positive role of PY residues in N-sh2 domain interaction. Whereas, LCK and HCK contain 23%, 27%, 25% and 29% conservation of Phe, Arg, Leu and Ser at + 1, +4, +2 and +6 positions, respectively in C-sh2 domain. The position of this PY pocket was then identified in crystal structure of Shp-2 and displayed in Rasmol prior to docking (Additional file 2A). The recognition of phosphorylated tyrosines in c-Kit by SH2 domain, and binding of these tyrosine residues to SH2 domain may occur in a typical "plug and socket" manner. It is similar to SH2 domain bound phosphotyrosine peptides complexes, as shown by NMR and X-ray structural studies [21,22].

### Docking of Shp-2 and c-Kit cytoplasmic domain

**Figure 3:**
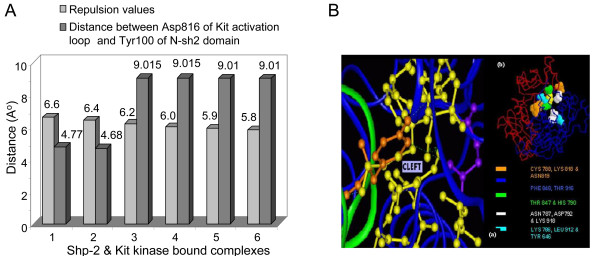
**Analysis of predicted interactive zones of Shp-2 and c-Kit**. **A**. Distances between interactive zones of both Shp-2 and Kit kinase based on different repulsion values. Shp-2 bound Kit kinase complexes are generated using different repulsion values from 5.8A° to 6.6 A°. Distance measured between Asp816 of Kit activation loop and Tyr100 of N-sh2 domain (as Tyr100 has been found to be prevalently closer to activation loop in all models). Among the 1-6 complexes, only 6.4A° and 6.6 A° based models have activation loop residue Asp816 closest to N-sh2 domain. **B**. Analysis of the catalytic cleft and the residues involved. Residues such as Cys788, Lys818 and Asn819 (orange), Thr916 and Phe 848 (blue), Thr847 and His790 (green), Asn787, Asp786 and Lys 918 (white), Lys786, Leu 912 and Tyr646 (mauve color), contribute in making a catalytic cleft like structure in c-Kit.

The protein docking study was performed using activated/phosphorylated crystal structure of Kit cytoplasmic domain and Shp-2 (which mimics interaction of ligand-dependent or independent activated Kit Kinase with Shp-1 *in vivo*) [8]. The bound ligand-substrate complexes were generated on the basis of different repulsion values used in the programme and the best chosen values were 6.6 and 6.4A° (Table 1). We also plotted different repulsion values against the distances measured between N-sh2 domain of Shp-2 and an activation loop residue Asp816 (Table 1 and Fig. 3A), as it plays crucial role in maintaining closed or open conformation of Kit kinase domain. The data showed that Ile571 and Val569 of "EEINGNNYV^569^YI ^571^DPTQ" came into close vicinity of Asp26 and Tyr100 of N-sh2 domain, whereas Asp816 stayed predominantly closer to Tyr100 of N-sh2 by 4.7A° (Additional file 2B, 3A, 3B). Thus we propose that there can be a possible mode of interaction between N-sh2 and activation loop residues containing Asp816, relevant to the modulation of conformation in activation loop.

**Table 1:** Residues found to be predominantly closer between N-sh2 and c-Kit

Repulsion values for docking (A°)	Residues found closer in Shp-2 bound Kit c-Kit: N-sh2	Distance observed c-Kit: N-sh2 (A°)	Domains interacting
6.6	Ile571-Asp26	8.59	Kit Juxtamembrane and PY pocket
6.6	Val569-Tyr100	7.07	Kit Juxtamembrane and N-sh2
6.6	Asp816-Tyr100	4.68	Kit activation loop and N-sh2
6.4	Ile571-Tyr100	5.66	Kit Juxtamembrane and N-sh2
6.4	Val569-Tyr100	4.46	Kit Juxtamembrane and N-sh2
6.6	Asp816-Tyr100	6.17	Kit activation loop and N-sh2

Repulsion values 6.6 and 6.4 A° show that conserved Juxtamembrane residues and activation loop residues are predominantly closer to N-sh2 domain residues.

### Checking of errors in docked complexes

Examining the substrate-bound complexes of Shp-2 is extremely essential while determining the distances between residues of interacting domains. The analysis showed negligible inter-atomic clashes, zero bumping into each other, and very few intramolecular constraints in Tyr100 of N-sh2. To verify hydrogen bonding, we changed the torsion angle from -156.66 to -116.66 between amino group of Tyr100 and the carboxyl group of Asn819, which showed that the hydrogen bonding of 4.76A° converted to 3.67A° (Additional file 3B).

### Analysis of protein-protein interaction

The most striking feature observed in Shp-2 bound Kit complex was the presence of a catalytic cleft like structure made of important residues, like Lys786 to Cys 788, Lys818, Asn819, His 790, Asp792, Thr847, Phe 848, Leu 912, Thr916, Lys 918 and Tyr646, which are highly conserved among c-Kit related kinases (Fig. 3B). These residues were found to be in very close proximity to Tyr100, Ala75, Gly76, Glu79, Met82, Gly83 and His85 of N-sh2, therefore expected to interact to form a stable conformation.

### Evaluation of hydrogen bonding between catalytic cleft and SH2 domains

**Figure 4:**
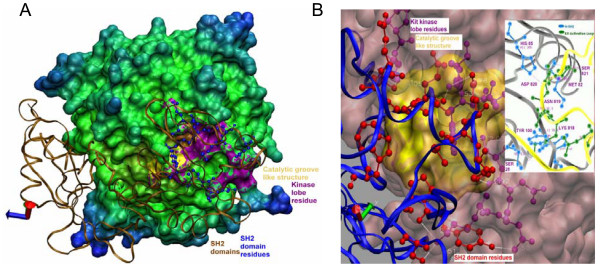
**Analysis of docked complexes and H-bond interactions between ligand and substrate (**2shp-1pgk.pdb). **A**. The docked complex show two SH2 domains as ribbons (pale golden color) and the blue color ball and stick structures show the residues of SH2 domains that interacted with Kit catalytic residues (stretch of violet color). The golden color displays the groove like structure in Kit catalytic domain. **B**. H-bonds are detected between SH2 domains and Kit cytoplasmic domain. Discontinuous white lines appear as the H-bonds between SH2 domain residues (red color ball and sticks) and Kit kinase lobe (pink color ball and sticks) residues. The white background displays important hydrogen bonding between Kit activation loop residues comprising Lys818 to Ser821 and N-sh2 domain residues.

Shp-2 bound activated Kit kinase complex mimics the physical interaction between autophosphorylated Kit kinase domain with the SH2 domains of Shp-1, both *in vitro *and *in vivo *(Fig 4A). We have detected that certain N-sh2 terminal residues, like Ser28, Ala75, Gly76, Glu79, Met82, Gly83, His85, Tyr100 and Pro101 (Fig. 4B, displayed in red colored balls and sticks) are making distinct hydrogen bonding with the c-terminal Kit kinase lobe residues (Fig. 4B, displayed in violet color balls and sticks). These results showed that residues from Lys818 to Ser821 in Kit catalytic cleft make distinct hydrogen bonding with both PY pocket and N-sh2 domain, for example Lys818 and Asn819 with Tyr100, Asp820 with His85, and Ser821 with Met82 (Fig. 4 and Table 2). Residues from 649-653 of N-terminal Kit kinase lobe were found to make hydrogen bonding with the C-sh2 domain residues. These interactions were detected specifically in surrounding of c-terminal kinase groove (Fig. 4B, in golden yellow) made of Leu783 as the tunnel-making residue inside groove. All the hydrogen bonding detected between conserved catalytic cleft and Kit kinase domains are at 2-3A° in distance. The earlier homology modeling study on Kit kinase has shown that release of auto-inhibition of activation loop is largely dependent on the disruption of intramolecular hydrogen bond interactions between Asp816 with the peptide backbones of Lys818 and Asn819, which transits the activation loop into a more flexible conformation [1].

**Table 2:** Hydrogen bonds detected between Kit kinase & SH2 domains

Positions of residues in substrate (Donor: c-Kit)	Position of residues in Ligand (Acceptor: Shp-1)	Distance of H-bonds in A°
649:ND2	192:O	2.54
649:ND2	193:O	1.12
650:N	192:O	2.03
650:ND1	195:OE1	2.11
650:ND1	195:OE2	2.07
650:NE2	195:OE2	2.98
652:N	195:OE1	1.44
652:N	195:OE2	2.64
653:N	195:OE1	2.56
655:N	200:OD1	2.04
656:N	200:OD1	2.86
671:OE2	196:O	2.27
671:OE2	199:O	2.66
775:NE2	213:O	2.18
782:N	3:OG	2.12
785:OG	3:OG	2.52
785:OG	103:OD1	2.89
802:N	211:OE1	2.27
803:N	211:O	2.68
807:NZ	197:O	2.51
807:NZ	198:O	2.53
**818:NZ**	**28:OG**	**2.51**
**818:NZ**	**100:O**	**2.83**
**819:ND2**	**100:OH**	**2.50**
**820:O**	**85:NE2**	**2.92**
**821:OG**	**82:O**	**2.03**
*847:OG1*	*83:OE1*	*2.83*
*847:OG1*	*83:OE2*	*2.09*
*848:N*	*83:OE2*	*2.84*
*849:N*	*83:OE2*	*2.19*
*849:OE2*	*79:O*	*2.58*
*849:OE2*	*83:OE1*	*2.49*
*849:OE2*	*83:OE2*	*1.91*
915:N	76:OE2	2.49
916:OG1	75:O	2.71

**A**: Represents hydrogen bonds between activation loop residues and N-sh2 domain residues *B*: Shows kinase lobe residues preceding c-terminal tail of c-Kit and N-sh2 domain residues

Our study suggest that subsequent loss of the above intramolecular hydrogen bonding, Lys818 and Asn819 of Kit activation loop might be interacting with conserved PY pocket residue (Ser28 and conserved Tyr100 of N-sh2 domain) for physical blocking of activation loop motifs for further interaction.

## Discussion

The activity of c-Kit is controlled through two important mechanisms: either by juxtamembrane segment auto-inhibition or by phosphatases mediated inhibition. The c-Kit is auto-inhibited by its juxtamembrane segment as it forms a V-shaped loop, inserted directly into the kinase lobes and displaces αC helix of Kit [23]. Subsequent to the activation of Kit kinase, the intramolecular H-bonds between Lys818-Asp816 and Asn819-Asp816 are disrupted, which further destabilizes the activation loop, releasing the steric hindrances associated with the rearrangement of the lobes. This phenomenon allows correct positioning of residues in and from active site, thus opening up the activated structure of Kit kinase (Additional file 4).

**Figure 5:**
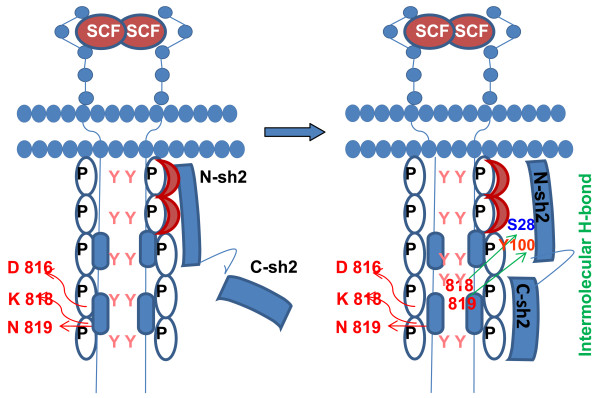
**Hypothesis generated on basis an *in silico *interaction of c-Kit (PDB: **1pgk) **and Shp-2 (2shp)**. N-sh2 domain of Shp-1 recognizes the dual-phosphorylated tyrosines at juxtamembrane (Y^568^V^569^Y^570^) by PY pocket. Shp-1 forms a stable complex with c-Kit kinase by making intermolecular H-bonds with Lys818 and Asn819, using Tyr 100 and Ser28 of N-sh2 domain, following disruption of the intramolecular hydrogen bonding of Asp816 with Lys818 and Asn819 in activated c-Kit.

On the basis of our investigation, we propose that in activated c-Kit, the residues Lys818 and Asn819 make intermolecular hydrogen bonding with N-sh2 domain residues "Tyr100 and Ser28" as a result of the disruption of intramolecular hydrogen bonding with Asp816 (Fig. 5). The constitutive change of Asp816 to Val may also lead to deleterious effects by disrupting these H-bonds with Lys818 and Asn819, as Asp816 has the capability to stabilize a positively charged α helical dipole by virtue of negative charge in its side chain and also serving as an amino-terminal capping to Ile817, Lys818, Asn819 and Asp820. So a point mutation to hydrophobic or aromatic amino acid would potentially destabilize this interaction, thereby forcing activation loop to transit from inactive to active confirmation, disrupting the Shp-1 association. This leads to an enhanced ubiquitinin-mediated degradation of Shp-1 [12]. Strikingly, Asp820 signature molecule of systemic mast cell disorder (SMCD) [represented as Asp820Gly] forms a hydrogen bond with the His85 of N-sh2 domain [24,25]. This also explains the role of Asp820 in Shp-1 binding. So it is considered that, Kit activation loop residues play a critical role in Shp-1 mediated negative regulation of Kit kinase.

## Conclusion

In summary, our study has revealed that certain activation loop residues in c-Kit are crucial for interaction of kinase with Shp-1, which may play a vital role in Shp-1 mediated down-regulation of c-Kit kinase activity. We propose that point mutation in Lys818 to Ser821 and Thr847 to Glu849 residues in Kit catalytic domain may lead to the generation of hyperactive functional variants of c-Kit. These may be implicated as efficient gene therapeutic candidates for conditional over-expression in the regeneration of hematopoietic compartments.

## Methods

### Multiple alignments of c-Kit

Using protein information resource (PIR) based multiple sequence alignment tool: PIR-ALN, a phylogenetic tree and multiple sequence alignment were generated for c-Kit and its kinase family members [26].

### Motifs scan of c-kit cytoplasmic domain

Motif Scan search identified short protein sequence in kinase domain of c-kit motifs that are either recognized by modular signaling domains, phosphorylated by protein Ser/Thr- or Tyr-kinases or mediate specific interactions with phospholipid ligands to initiate c-kit mediated cell specific signaling [27].

### Detection of phosphotyrosine binding motifs in SH2 domains of tyrosine phosphatases

The SH2 domain containing proteins were aligned using CLUSTALW for detecting recognition and binding motif in Shp-2, which initiate recruitment of SH2 domains to Kit kinase [28].

### Docking study on c-Kit and Shp-2 interactions and visualization

We have used GRAMM (Global Range Molecular Matching) program to generate Shp-2 bound Kit kinase complexes and predicted H-bond interactions between them. The principle and the methodology of GRAMM are available at the web address http://vakser.bioinformatics.ku.edu/resources/gramm/gramm1/install/readme.pdf. Different repulsion values were applied during docking to determine the best fit complexes (Fig. 3). These repulsion values in A° were determined by three factors, r_ij _(distance between ligand and receptor), U (energy of repulsion) and R, "range" of the potential (the grid step) using the mathematical relationship [29,30], as shown in the Additional file 5. Distances lesser than 10A°, between important residues (within the predicted interactive zones), decided the best positioning of two proteins for further analysis (Fig. 3). We have used crystal structure of Shp-2 (protein tyrosine phosphatase), 2shp, a full length structural relative of Shp-1, solved at a resolution of 2A°) [13] and activated crystal structure of human c-Kit cytoplasmic domain (PDB: 1pkg; solved at a resolution of 2.9A° as ligand and substrate) [5]. Analysis of models for the number of inter/intra-molecular clashes, formation of hydrogen bonding and visualization of model images were done by VMD programme.

## List of abbreviations

**HCK**: Hematopoietic cell kinase; **LCK**: Lymphocyte Specific Protein Tyrosine Kinase p56; **PY**: Phosphotyrosine binding pocket; **GRB-2**: Growth factor receptor bound protein 2; **PSSM**: Position-specific scoring matrix; **GRAMM**: Global Range Molecular Matching; **SCF**: Stem cell factor; Shp-1/2: Tyrosine phosphatase-1/2; **Src**: Sarcoma proto-oncogene; **c-ZAP**: zeta-chain [TCR] associated protein kinase 70 kDa; **JMD**: Juxtamembrane domain; **APS**: adapter protein with Pleckstrin homology and Src homology two domain.

## Competing interests

The authors declare that they have no competing interests.

## Authors' contributions

SP: conceptualization and designing the objectives of the paper, *in silico *protein interaction study, multiple alignments, analysis and interpretation of data and initial drafting of the manuscript. GUG: strategic planning to conduct *in silico *experiments in the initial phase of the study and revising the manuscript. OPK: revising the manuscript and final approval. AM: analysis and interpretation of data and revising the manuscript critically for important intellectual content.

All authors have read the final version of the manuscript and approved for the submission.

## Supplementary Material

Additional file 1 **Figure S1**. Motif Scan analysis of c-Kit juxtamembrane with other kinase relatives. Tyr568 and Tyr570 of YVY motif in c-Kit juxtamembrane domain are found to be involved in interaction with different molecules such as EGFR kinase and CRKL binding domain, as detected by motif scan. Click here for file

Additional file 2 **Figure S2**. Analysis of Shp2 bound and unbound Kit kinase structures. (A) The unbound form of Shp-1 displayed two sh2 domains and PY pocket. (B&C) The conserved domains like N/C-sh2, PY pocket, Y^568^V^569^Y^570 ^and residue Tyr100 of N-sh2 domain, found to be closer within an interactive zone, are displayed both in unbound and bound structures of c-Kit in different colors. Click here for file

Additional file 3 **Figure S3**. Detection of a catalytic groove like structure in Kit kinase and validation of hydrogen bonding. (A) A distinct groove like structure was found in the Shp2-Kit bound complex (shown within a square in the center). (B) The cleft also show one H-bond between N-sh2 domain and residue Tyr100 and Asn819 of Kit activation loop. Change in torsion angle from -156.66 to -116.66 show the same hydrogen bonding between the cleft and Tyr100 in a closer distance of 3.67A°. Click here for file

Additional file 4 **Figure S4**. Interaction of c-Kit with various down-stream signaling molecules. Lys818 and Asn819 make intramolecular hydrogen bonding with Asp816 in inactive c-Kit, making a closed conformation of c-Kit kinase. Upon binding of SCF, a chain of molecular events take place, comprising disruption of above intramolecular H-bonds and recruitment of tyrosine kinases and tyrosine phosphatases at the respective phosphorylated tyrosine containing docking sites at c-Kit catalytic domain. Click here for file

Additional file 5 **Mathematical relationship**. The method for calculating ligand-receptor interaction energy Click here for file
