# Supplementary figures and images for: The structural insights of stem cell factor receptor (c-Kit) interaction with tyrosine phosphatase-2 (Shp-2): An in silico analysis
Source: BMC Res Notes. 2010 Jan 22;3:14. doi: 10.1186/1756-0500-3-14 (PMC2826351; doi:10.1186/1756-0500-3-14)

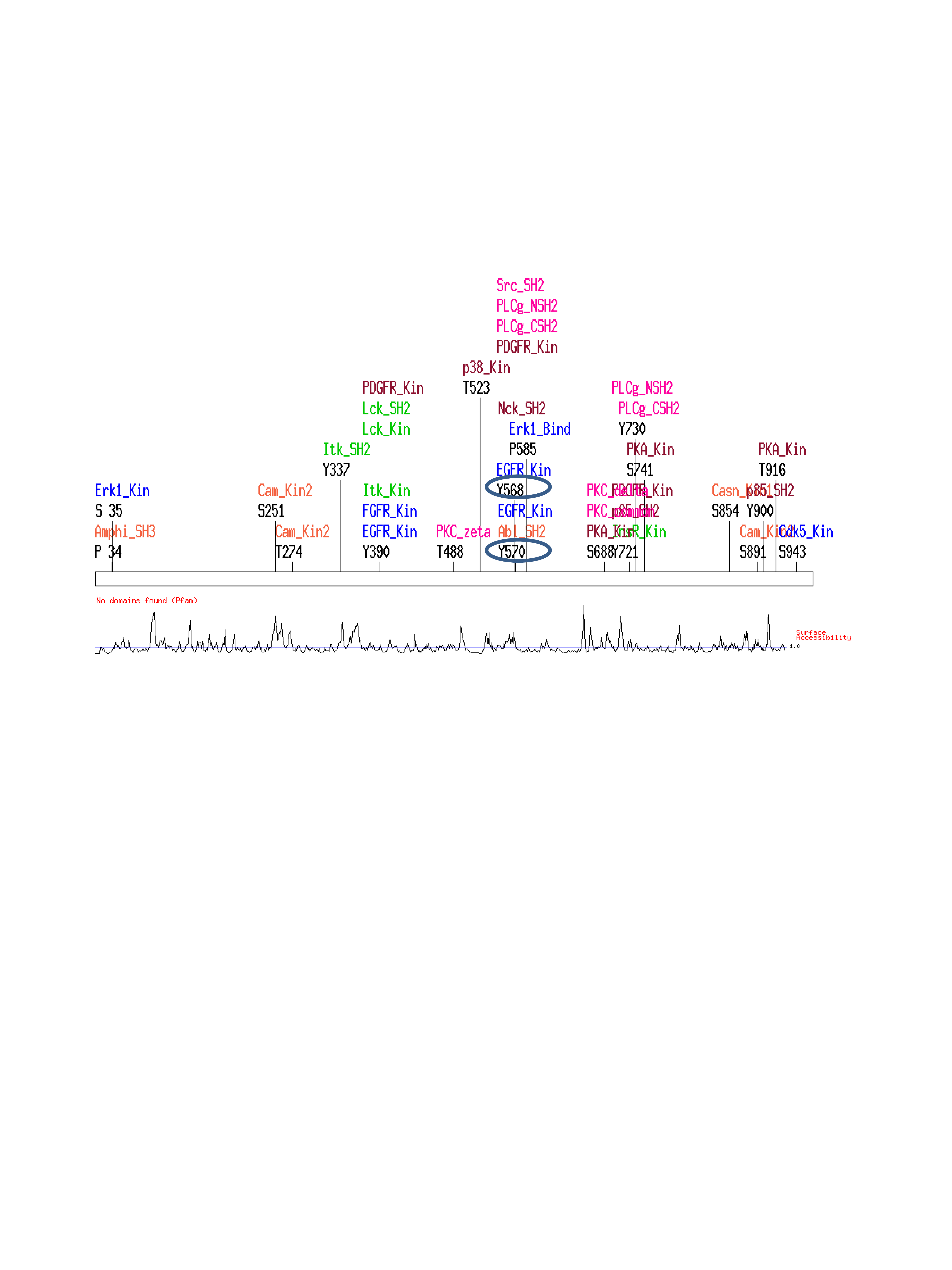

Supplement: Additional file 1 — Figure S1. Motif Scan analysis of c-Kit juxtamembrane with other kinase relatives. Tyr568 and Tyr570 of YVY motif in c-Kit juxtamembrane domain are found to be involved in interaction with different molecules such as EGFR kinase and CRKL binding domain, as detected by motif scan. [file 1756-0500-3-14-S1.TIFF]

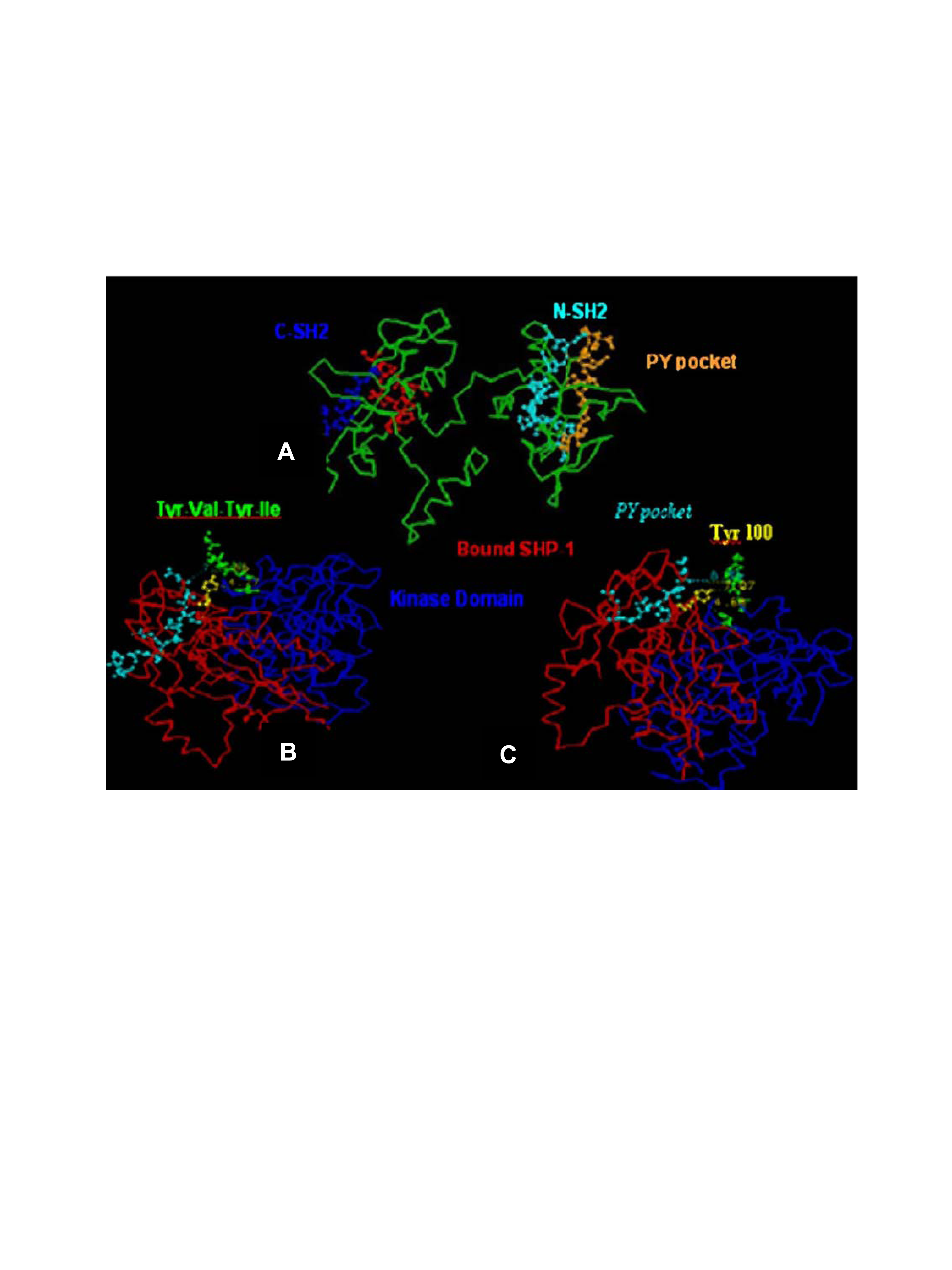

Supplement: Additional file 2 — Figure S2. Analysis of Shp2 bound and unbound Kit kinase structures. (A) The unbound form of Shp-1 displayed two sh2 domains and PY pocket. (B&C) The conserved domains like N/C-sh2, PY pocket, Y568V569Y570 and residue Tyr100 of N-sh2 domain, found to be closer within an interactive zone, are displayed both in unbound and bound structures of c-Kit in different colors. [file 1756-0500-3-14-S2.TIFF]

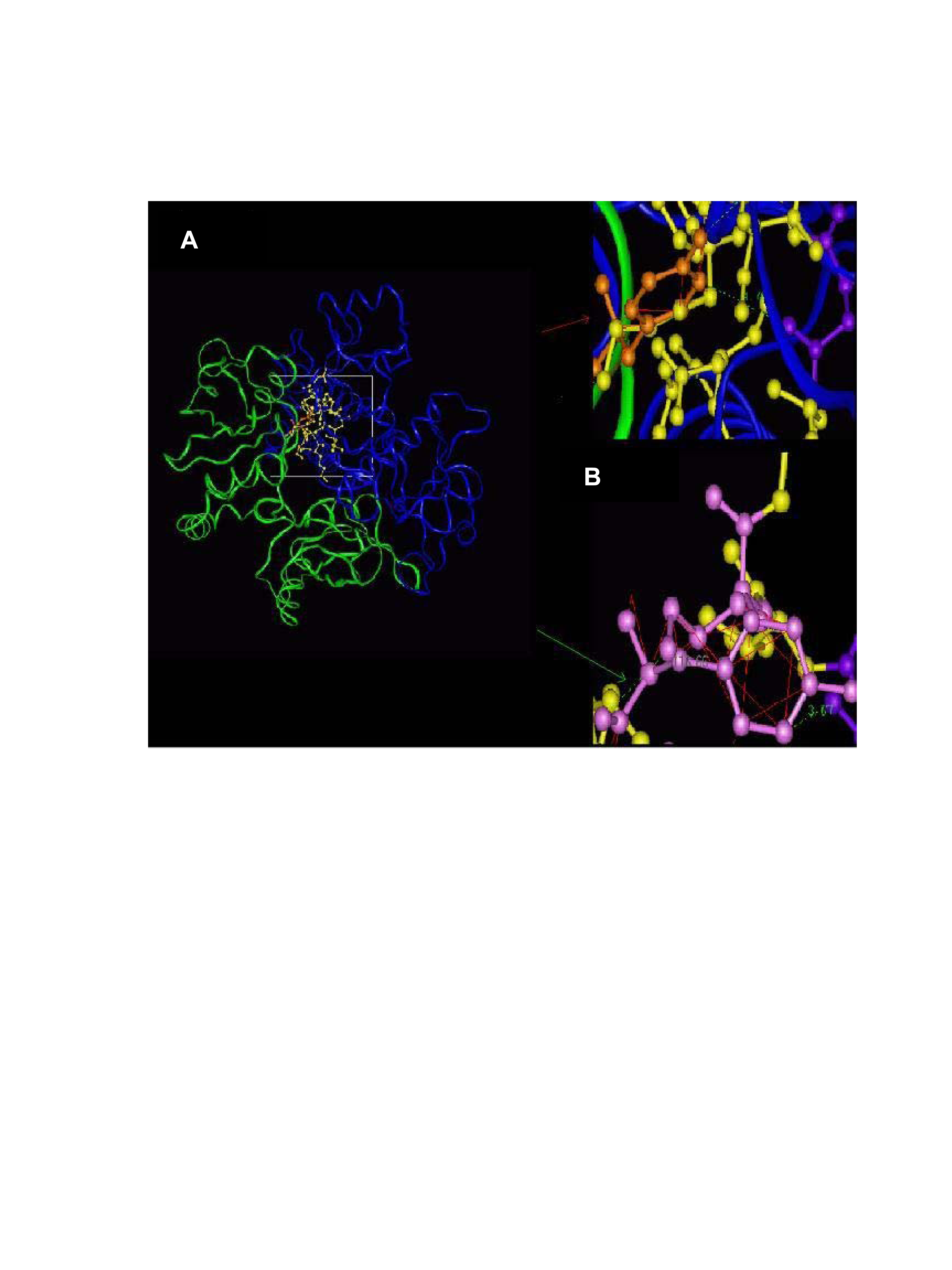

Supplement: Additional file 3 — Figure S3. Detection of a catalytic groove like structure in Kit kinase and validation of hydrogen bonding. (A) A distinct groove like structure was found in the Shp2-Kit bound complex (shown within a square in the center). (B) The cleft also show one H-bond between N-sh2 domain and residue Tyr100 and Asn819 of Kit activation loop. Change in torsion angle from -156.66 to -116.66 show the same hydrogen bonding between the cleft and Tyr100 in a closer distance of 3.67A°. [file 1756-0500-3-14-S3.TIFF]

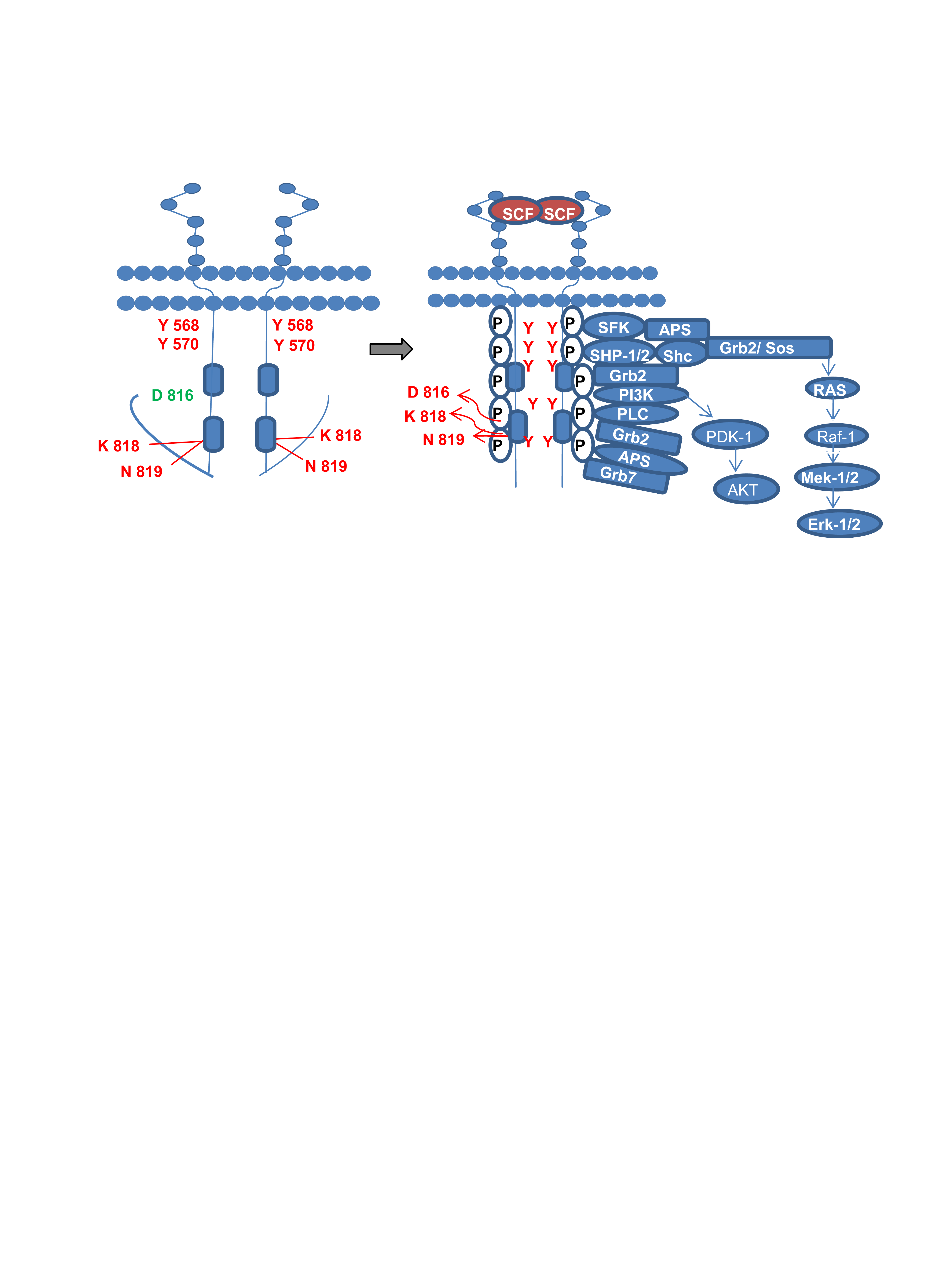

Supplement: Additional file 4 — Figure S4. Interaction of c-Kit with various down-stream signaling molecules. Lys818 and Asn819 make intramolecular hydrogen bonding with Asp816 in inactive c-Kit, making a closed conformation of c-Kit kinase. Upon binding of SCF, a chain of molecular events take place, comprising disruption of above intramolecular H-bonds and recruitment of tyrosine kinases and tyrosine phosphatases at the respective phosphorylated tyrosine containing docking sites at c-Kit catalytic domain. [file 1756-0500-3-14-S4.TIFF]

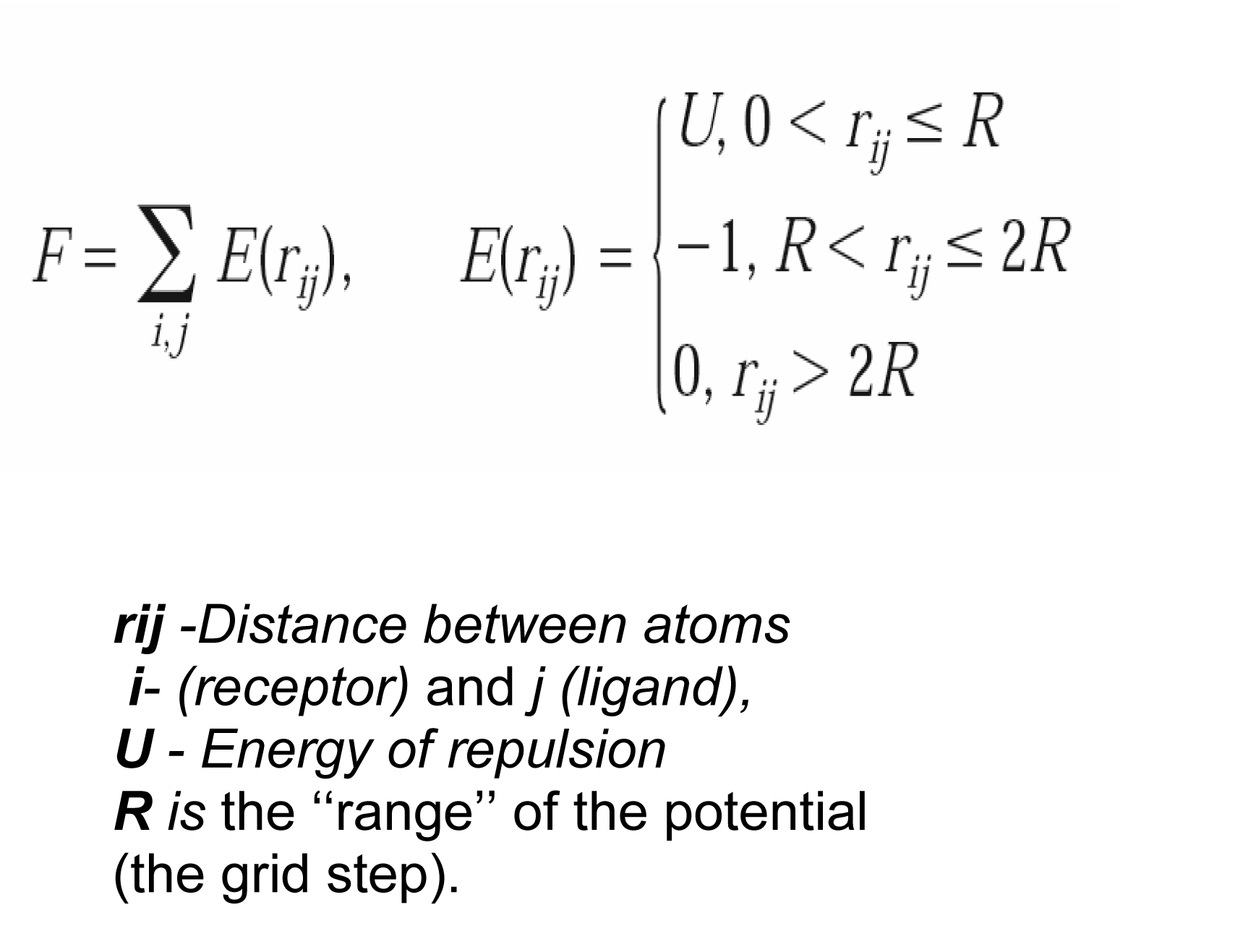

Supplement: Additional file 5 — Mathematical relationship. The method for calculating ligand-receptor interaction energy [file 1756-0500-3-14-S5.TIFF]
